# Role of liensinine in sensitivity of activated macrophages to ferroptosis and in acute liver injury

**DOI:** 10.1038/s41420-023-01481-3

**Published:** 2023-06-23

**Authors:** Jing Li, Qi Huang, Minling Lv, Wenfeng Ma, Jialing Sun, Xin Zhong, Rui Hu, MengQing Ma, Zhiyi Han, Wei Zhang, Wenxing Feng, Xinfeng Sun, Xiaozhou Zhou

**Affiliations:** 1 Department of Liver Disease, The Fourth Clinical Medical School, Guangzhou University of Chinese Medicine, Shenzhen, 518033 China; 2 Department of Liver Disease, Shenzhen Traditional Chinese Medicine Hospital, Shenzhen, 518033 China; 3 Macau University of Science and Technology, Faculty of Chinese Medicine, Taipa, Macao, 999078 China

**Keywords:** Diseases, Health sciences

## Abstract

Acute liver injury (ALI) is an acute inflammatory liver disease with a high mortality rate. Alternatively, activated macrophages (AAMs) have been linked to the inflammation and recovery of ALI. However, the mechanism underlying AAM death in ALI has not been studied sufficiently. We used liensinine (Lie) as a drug of choice after screening a library of small-molecule monomers with 1488 compounds from traditional Chinese remedies. In ALI, we evaluated the potential therapeutic effects and underlying mechanisms of action of the drug in ALI and found that it effectively inhibited RSL3-induced ferroptosis in AAM. Lie significantly reduced lipid peroxidation in RSL3-generated AAM. It also improved the survival rate of LPS/D-GalN-treated mice, reduced serum transaminase activity, suppressed inflammatory factor production, and may have lowered AAM ferroptosis in ALI. Lie also inhibited ferritinophagy and blocked Fe^2+^ synthesis. Following combined treatment with RSL3 and Lie, super-resolution microscopy revealed a close correlation between ferritin and LC3-positive vesicles in the AAM. The co-localization of ferritin and LC3 with LAMP1 was significantly reduced. These findings suggest that Lie may ameliorate ALI by inhibiting ferritinophagy and enhancing AMM resistance to ferroptosis by inhibiting autophagosome-lysosome fusion. Therefore, Lie may be used as a potential therapeutic agent for patients with ALI.

## Introduction

Acute liver injury (ALI), also known as acute liver failure, is caused by various factors, including drug stimulation [1], viral infection [2], alcohol consumption [3], and chemical toxins [4]. It is associated with severe impairment or liver function loss. Further, ALI is characterized by its rapid onset, severe disease, and several other complications [5]. Unfortunately, no specific drugs or treatment approaches for ALI are available [6], and artificial liver support treatment cannot completely restore liver function due to large-scale liver cell necrosis [7]. Therefore, the sole viable treatment option is liver transplantation. However, due to the lack of suitable donor tissue, the associated life-long immunosuppression, high costs, and technical limitations, it is not an ideal treatment option [8]. Therefore, developing safe drugs with effective mechanisms of action and enhanced therapeutic outcomes for patients with ALI is crucial.

Liver inflammation plays a significant role in the etiology of ALI [9]. Macrophages have innate immune and paracrine functions; hence, an imbalance in their function can lead to ALI-related liver inflammation progression or resolution [10–12]. Based on their activity, macrophages are widely categorized into two phenotypes: classical macrophages (M1) and alternatively activated macrophages (AAM) [13]. Intrahepatic macrophages in ALI are mainly M1 macrophages, which synthesize and secrete various inflammatory factors, such as interleukin (IL)-1β and IL-6, accelerating liver inflammation progression [14–16]. AAM macrophages play a hepatoprotective role in ALI [11, 17, 18], such as protecting the liver in acute and chronic liver failure by inhibiting the necroptosis-S100A9-necroinflammatory axis [19]. AAM macrophages reduced hepatocyte necrosis and neutrophil infiltration while reducing liver inflammation in an APAP-ALI experimental model [20]. Therefore, the balance between M1 macrophages and AAM may be an effective therapeutic target for ALI. ALI causes significant damage and death of intrahepatic cells, including hepatocytes and macrophages [21]. It is unknown whether M1 macrophages and AAM have different mortality patterns, which could affect their balance. Oxidative stress results in several cell death patterns [22–25]. Owing to the variable expression of antioxidant-related proteins, including inducible nitric oxide synthase (iNOS), M1 macrophages and AAM have differing antioxidative defense capabilities [26]. Therefore, we hypothesized that M1 macrophages and AAM would be differentially sensitive to one type of oxidative stress-related cell death, potentially resulting in an imbalance between both phenotypes and exacerbating liver inflammation progression in ALI. Elucidating the potential differential death mechanisms of M1 macrophages and AAM could lead to a novel strategy for developing effective drugs for treating ALI.

Ferroptosis is a newly discovered oxidative, non-apoptotic form of cell death that differs in morphology, biochemistry, and genetics from other types of cell death [27]. Ferroptosis has been linked to various liver diseases, including drug-induced liver injury and nonalcoholic fatty liver disease [28–30]. Macrophages express key genes involved in iron uptake, processing, export, and lipid metabolism [31]. Several studies have revealed that inhibiting macrophage ferroptosis can reduce pulmonary inflammatory injury in acute lung injury. However, the underlying mechanisms are unknown [32]. During ALI pathogenesis, M1 macrophages dominate intrahepatic macrophages [14], and the number of AAM is lower than normal levels [17]. AAMs are also highly sensitive to ferroptosis [33]. Therefore, we hypothesized that reactive oxygen species (ROS) accumulation during ALI pathogenesis triggers AAM ferroptosis, reduces AMM numbers, and disrupts the balance between M1 macrophages and AAM, aggravating the hepatic inflammatory injury. Therefore, inhibiting AAM ferroptosis in ALI would be beneficial in restoring macrophage phenotype balance.

Traditional Chinese medicine (TCM) is a result of the traditional culture and wisdom of China. TCMs have been linked to fewer toxic side effects, several therapeutic targets, and significant therapeutic efficacy. Natural product bioactive components are important drug sources and have the potential to be used in drug screening [34]. Several TCMs have been found to influence ferroptosis regulation [35] significantly. Therefore, developing treatment for ALI by identifying novel medications from TCM monomer molecules is feasible. A mouse model of lipopolysaccharide (LPS)/D-GalN-induced ALI was used to determine M1 macrophages and AAM sensitivity to ferroptosis. TCM monomer compound library was used to screen for and assess small-molecule compounds that can effectively inhibit AAM ferroptosis and evaluate their potential therapeutic value. This study established a scientific basis for research and development of hepatoprotective drugs against AAM ferroptosis and provides novel insights into the mechanism of Lie against ALI.

## Results

### Sensitivity of alternatively activated macrophages to lipid peroxidation-driven ferroptosis

**Fig. 1:**
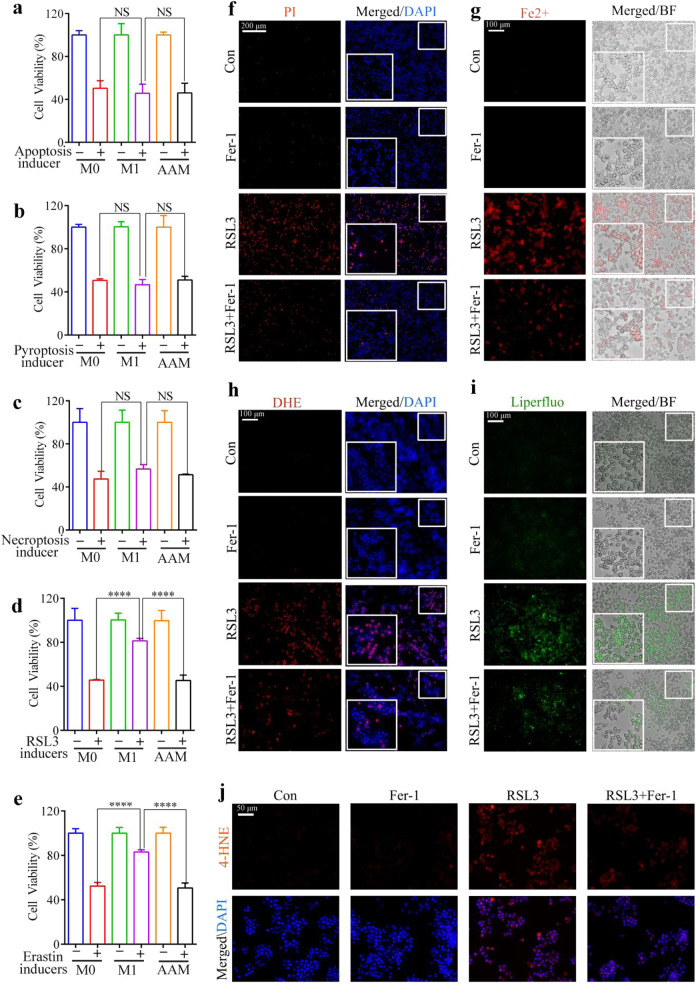
Sensitivity of alternatively activated macrophages to lipid peroxidation-driven ferroptosis. **a**–**e** CCK-8 assay for cell viability after the treatment of macrophages (M0, M1, AAM) with the apoptosis inducer (20 μM, 24 h), pyroptosis inducers (7 μg/ml, 24 h), necrosis inducer (4×, 24 h), RSL3 inducer (5 μM, 5 h), and erastin inducer (60 μM, 24 h). **f**–**i** AAM was treated with RSL3 (5 μM, 5 h) in the presence or absence of Fer-1 (400 nM). **f** PI staining to assess cell death, Scale bar = 200 µm. **g** Live cell fluorescence imaging to detect lipid peroxide production using Liperfloo, Scale bar = 100 µm. **h** Live cell fluorescence imaging of FerroOrange (red), Scale bar = 100 µm. **i** Fluorescence imaging of the superoxide anion fluorescence detection probe Dihydroethidium (DHE), Scale bar = 100 µm. **j** Expression of the 4-hydroxynonenal (4-HNE) protein was measured by immunofluorescence, Scale bar = 50 µm. Results were presented as mean ± SD. (*n* = 3, ****p < 0.0001. Con control, NS not significant).

We assessed the sensitivity of different types of macrophages to determine different forms of cell death. Unstimulated RAW264.7 macrophages were polarized to the AAM or M1 macrophage states after treatment with IL-4 or LPS/IFN-γ, respectively (Fig. S1a). The M0 macrophages, M1 macrophages, and AAM were then treated with the appropriate IC_50_ doses of apoptosis, pyroptosis, ferroptosis, and necrosis inducers (Fig. S1b–f) to observe cell viability changes. The ability of the apoptosis, pyroptosis, and necrosis inducers to inhibit cell viability in the M0 macrophages, M1 macrophages, and AAM did not differ significantly (Fig. 1a–c). However, RSL3, a ferroptosis inducer, significantly inhibited M0 macrophages and AAM viability compared to that of the M1 macrophages (Fig. 1d). Further, similar results were observed when the M0 macrophages, M1 macrophages, and AAM were treated with erastin (a ferroptosis inducer) (Fig. 1e). We then treated AAM with Fer-1 combined with RSL3 and found that Fer-1 could reverse RSL3-induced AAM death (Fig. 1f), reduce Fe^2+^ (Fig. 1g) and ROS levels (Fig. 1h), and inhibit intracellular lipid oxidation product generation (Fig. 1i, j). These findings suggest that AAMs are ferroptosis-sensitive, whereas M1 macrophages are ferroptosis-resistant.

### Relationship between Fer-1 treatment of LPS/D-GalN-induced liver injury in mice and alternatively activated macrophages

**Fig. 2:**
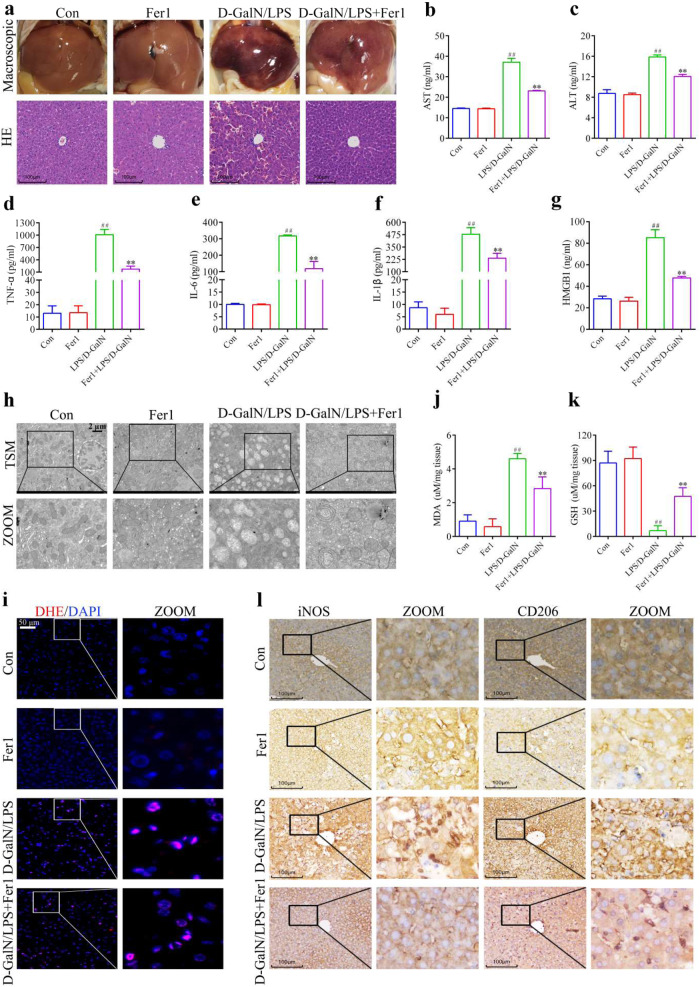
Fer-1 attenuates liver injury induced by LPS/D-GalN in mice, accompanied by increased numbers of alternatively activated macrophages. **a** Liver injury was assessed using histopathology and hematoxylin and eosin (H&E) staining, Scale bar = 100 µm. **b**, **c** The levels of AST and ALT in serum. **d**–**g** Serum content of TNF-α, IL-6, IL-1β, and HMGB1. **h** Transmission electron microscopy (TEM) shows a representative liver tissue image, Scale bar = 2 μm. **i** Representative fluorescence imaging of the superoxide anion fluorescence detection probe dihydroethidium (DHE) in liver tissue, Scale bar = 50 µm. **j**, **k** Assessment of MDA and GSH contents in liver tissue. **l** Expression of iNOS and CD206 was detected using immunocytochemistry in the liver tissue, Scale bar = 100 µm. Results were presented as mean ± SD (*n* = 5). (^##^*p* < 0.01, vs. Con. **p < 0.01, vs. D-GalN/LPS. Con Control, NS not significant).

We then explored the role of ferroptosis in ALI. We examined the LPS/D-GalN-induced histopathological changes in the liver tissues of Fer-1 treated and untreated mice and examined serum transaminases and inflammatory factor levels. The histological analysis and H&E staining results indicated that Fer-1 alleviated histopathological changes in the livers of the LPS/D-GalN-induced mice, including inflammatory infiltration, disorganization, and hepatocyte swelling (Fig. 2a). Furthermore, results of the serum transaminases and inflammatory factors using ELISA showed that when the mice were injured by LPS/D-GalN treatment, AST (Fig. 2b), ALT (Fig. 2c), and inflammatory factors (Fig. 2d–g) increased significantly but decreased significantly in the Fer-1 + LPS/D-GalN Group mice (Fig. 2b–g). In addition, in the LPS/D-GalN-treated mice, Fer-1 partially reversed liver mitochondrial damage (Fig. 2h) and inhibited ROS production (Fig. 2i). Furthermore, MDA and 4-HNE production were reduced by Fer-1 combined with LPS/D-GalN-treated mice compared to LPS/D-GalN-treated mice (Figs. 2j and S2), where GSH production was elevated (Fig. 2k). Immunohistochemistry was used to further examine changes in M1 macrophages and AAM levels. In LPS/D-GalN-treated mice, the M1 macrophage marker (iNOS) level significantly increased, whereas that of CD206 of AAM significantly decreased, which was partially reversed by the Fer-1 treatment (Fig. 2l).

### Screening TCM library for active compounds against alternatively activated macrophages ferroptosis

**Fig. 3:**
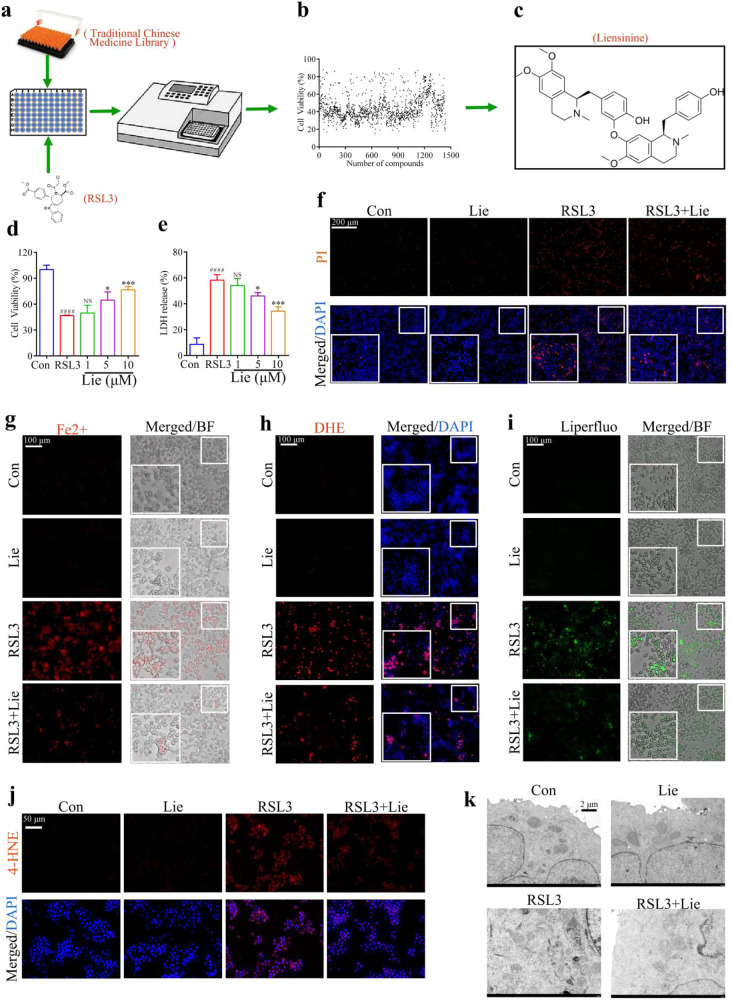
Use of the traditional Chinese medicine liensinine against alternatively activated macrophage ferroptosis in vitro. **a** Schematic of the drug-screening program. **b** AAM was treated with the 1488 candidates in combination with RSL3 (5 μM) for 5 h, and cell viability was measured using the CCK-8 assay kit. Each point represents the percentage of cell viability for a concentration of 10 μM of the candidate compound. **c** Chemical structure diagram of liensinine (Lie). **d** CCK-8 assay for cell viability. **e** Amount of LDH in the cell supernatant. **f** PI staining to assess cell death, Scale bar = 200 µm. **g** Live cell fluorescence imaging of FerroOrange (red), Scale bar = 100 µm. **h** Fluorescence imaging of the superoxide anion fluorescence detection probe dihydroethidium (DHE), Scale bar = 100 µm. **i** Live cell fluorescence imaging using Liperfloo, Scale bar = 100 µm. **j** Expression of the 4-HNE protein was measured by immunofluorescence, Scale bar = 50 µm. **k** Representative electron micrograph image of cells, Scale bar = 2 μm. Results were presented as mean ± SD. (*n* = 3, ^####^*p* < 0.0001, vs. Con. *p < 0.05, ***p < 0.001, vs. RSL3. Con Control, NS not significant).

In ALI, AAM plays a crucial role in repairing and inhibiting inflammation [10]; therefore, promoting AAM survival may be a promising therapeutic strategy. We screened the TCM library for small-molecule compounds that could inhibit ferroptosis in AAM. Cell viability was examined by treating AAM for 5 h with 1488 monomers obtained from the TCM library combined with RSL3 (Fig. 3a). Figure 3b shows that Lie was the most effective product. However, the Lie treatment did not significantly affect the M0 macrophages, M1 macrophages, or AAM (Fig. S3a–d); Fig. 1c depicts its chemical structure. Its effect on RSL3-induced ferroptosis in AAM was evaluated by treating the cells with varying concentrations (1, 5, and 10 μM) of Lie combined with RSL3. Lie increased cell viability in a dose-dependent manner (Fig. 3c). In addition, LDH level decreased in a dose-dependent manner (Fig. 3d). A Lie concentration of 10 μM was the most effective dose that promoted the survival of the AAM. The PI staining results showed that AAM treated with 10 μM Lie had a significantly lower RSL3-induced death rate (Fig. 3f). Immunofluorescence results revealed that Lie inhibited the synthesis of Fe^2+^ in the RSL3-treated AAM (Fig. 3g) and reduced the production of intracellular ROS (Fig. 3h) and lipid oxidation products (Fig. 3i, j). The electron microscopy results showed that exposure to Lie and RSL3-treated AAM reduced the number of damaged mitochondria and those with reduced or missing mitochondrial cristae (Fig. 3k). Lie also partially reversed erastin-induced ferroptosis in AAM (Fig. S3e–g).

### In vivo attenuation of liver injury and the inflammatory response using liensinine

**Fig. 4:**
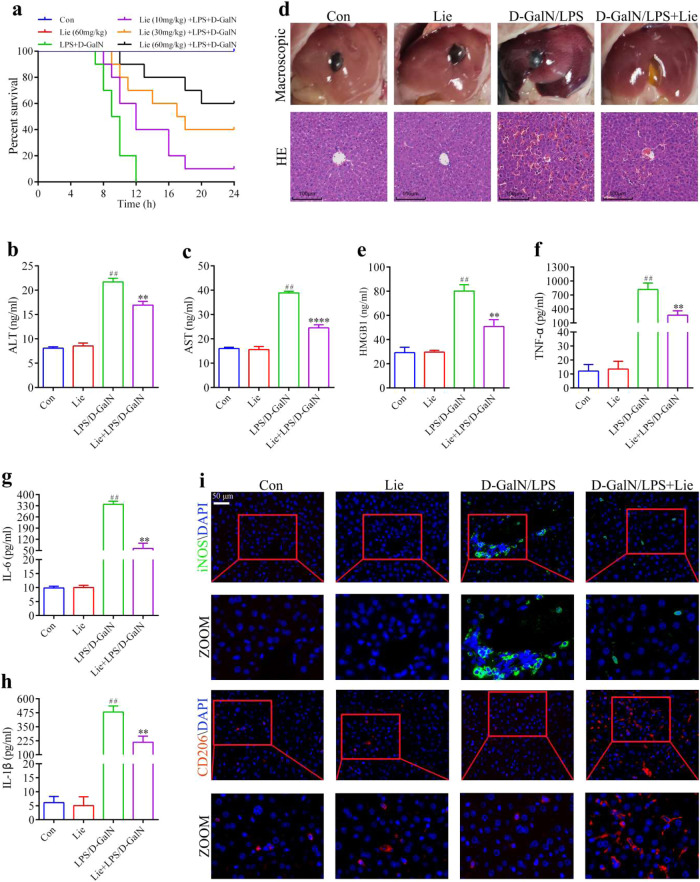
Liensinine treatment attenuates LPS/D-GalN-induced pathological liver injury and inflammatory responses in mice. **a** Survival was monitored over a 24 h period (*n* = 10/group). **b**, **c** Serum ALT and AST levels. **d** Histopathology, hematoxylin, and eosin (H&E) staining, Scale bar = 100 µm. **e**–**h** Determination of the levels of the inflammatory factors HMGB1, TNF-α, IL-6, and IL-1β in mice sera. **i** Expression of iNOS and CD206 in liver tissue was detected by immunofluorescence, Scale bar = 50 µm. Results were presented as mean ± SD (*n* = 5). (^##^*p* < 0.01, vs. Con. *p < 0.05, **p < 0.01, ****p < 0.0001, vs. LPS/D-GalN. Con control, NS not significant).

We examined LPS/D-GalN-induced survival in Lie-treated and untreated mice to determine whether Lie may offer ALI protection in vivo. Findings revealed that LPS/D-GalN caused a high mortality rate, with 100% mortality occurring within 24 h. Lie treatment increased survival in a dose-dependent manner. For example, 60% of the Lie-treated (60 mg/kg) mice group survived after 24 h (Fig. 4a). We then examined the effects of Lie on serum transaminase levels in LPS/D-GalN-treated mice. Our findings revealed that Lie significantly inhibited the activities of ALT and AST in the mice sera (Fig. 4b, c). H&E staining results revealed that Lie partially alleviated LPS/D-GalN-induced hepatotoxicity (Fig. 4d). The ELISA results showed that Lie treatment significantly reduced serum TNF-α, IL-6, HMGB1, and IL-1β levels in the LPS/D-GalN-treated mice (Fig. 4e–h). Furthermore, the administration of Lie alone had no significant effect on the liver tissue or inflammatory cytokine levels (Fig. 4a–h), implying that the Lie doses used in this study had no adverse effects on the liver (Fig. 4d). We also used immunofluorescence to examine the changes in the M1 macrophage and AAM content. Lie treatment significantly inhibited iNOS expression while increasing CD206 expression (Fig. 4i).

### In vivo possible inhibition of alternatively activated macrophage ferroptosis using liensinine

**Fig. 5:**
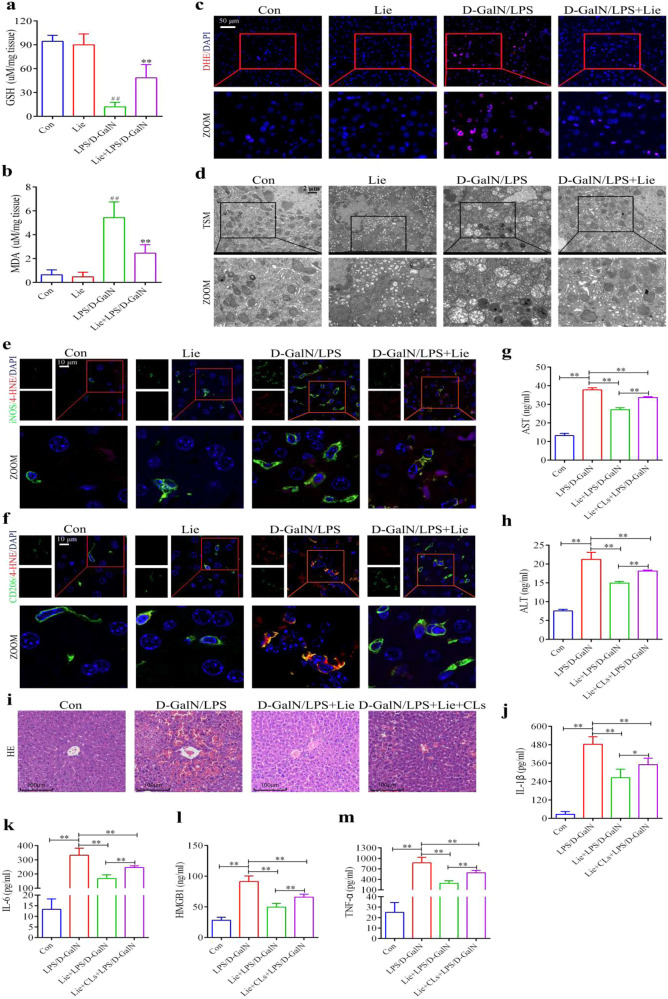
Liensinine inhibits LPS/D-GalN-induced alternatively activated macrophage ferroptosis in mice. **a**, **b** Assessment of GSH and MDA contents in liver tissues. (^##^*p* < 0.01, vs. Con. **p < 0.01, vs. LPS/D-GalN. Con Control). **c** Superoxide anion fluorescence detection probe dihydroethidium (DHE) was used to assess the level of ROS in the liver, Scale bar = 50 µm. **d** Representative electron micrograph of liver tissue, Scale bar = 2 µm. **e** Fluorescence analysis showing the co-localization of iNOS (green) with 4-HNE (red) in the liver, Scale bar = 10 µm. **f** Fluorescence analysis showing the co-localization of CD206 (green) with 4-HNE (red) in the liver, Scale bar = 10 µm. **f**–**h** Macrophages were depleted by intraperitoneal injections of clodronate liposomes (CLs) for 48 h. Mice-depleted macrophages were treated with Lie (60 mg/kg) intraperitoneally for 2 h and then injected with LPS/D-GalN for 6 h, after which the liver and blood were collected for subsequent experiments (*n* = 5/group). **f** Histopathology, hematoxylin, and eosin (H&E) staining, Scale bar = 100 µm. **g**, **h** Assessment of GSH and MDA contents in the mice sera. **i**–**m** Assessment of serum inflammatory factors IL-1β, IL-6, HMGB1, and TNF-α. Results were presented as mean ± SD (*n* = 5). (*p < 0.05, **p < 0.01. Con control).

Ferroptosis plays an important role in ALI, and Lie can partially inhibit RSL3-induced ferroptosis in AAM in vitro. Owing to the fact that Lie also increased the number of AAM in the LPS/D-GalN-induced mice, we hypothesized that Lie could increase the number of AAM in ALI by inhibiting ferroptosis. Lie partially reversed the decreased GSH content in LPS/D-GalN-induced mice (Fig. 5a), partially inhibited MDA production (Fig. 5b), and inhibited ROS production (Fig. 5c). We also used TEM to investigate mitochondrial morphology and found that the mitochondrial cristae were reduced or even absent in the livers of the LPS/D-GalN-treated group, the outer mitochondrial membrane was ruptured, and Lie treatment partially reversed the LPS/D-GalN-induced mitochondrial damage in the liver (Fig. 5d). These findings revealed that Lie could be used to treat ferroptosis induced by LPS/D-GalN. Immunofluorescence results showed that 4-HNE expression was significantly increased in the CD206-positive cells in liver tissues of LPS/D-GalN-treated mice, and it was lower in the iNOS-positive cells. In the Lie- and LPS/D-GalN-treated mice, the iNOS-positive cells and 4-HNE production in the CD206-positive cells were significantly reduced.

We further assessed whether the effect of Lie in inhibiting LPS/D-GalN-induced pathological liver injury and inflammatory responses in mice was partly attributed to AAM. Mice were depleted of macrophages using intraperitoneal injections of clodronate liposomes, followed by LPS/D-GalN injections. H&E staining of samples revealed that the effects of Lie on LPS/D-GalN-induced pathological liver injury in the macrophage-depleted mice were significantly reduced (Fig. 5f). Furthermore, clodronate liposomes were found to be effective in LPS/D-GalN-treated mice as they partially reversed the effects of Lie on serum transaminases (Fig. 5g, h). Furthermore, the efficacy of Lie to suppress hepatic inflammatory responses in the LPS/D-GalN-treated mice was significantly reduced in macrophage-depleted mice (Fig. 5i–m). However, Lie partially inhibited ROS and 4-HNE production in the liver of macrophage-depleted mice (Fig. S4a, b), suggesting that Lie partially ameliorates LPS/D-GalN-induced hepatocyte ferroptosis. These findings suggest that the effects of Lie on LPS/D-GalN-induced pathological liver injury and inflammatory responses in mice are partially related to AAM survival.

### Inhibition of ferritinophagic flux by liensinine increases resistance of alternatively activated macrophages to ferroptosis

**Fig. 6:**
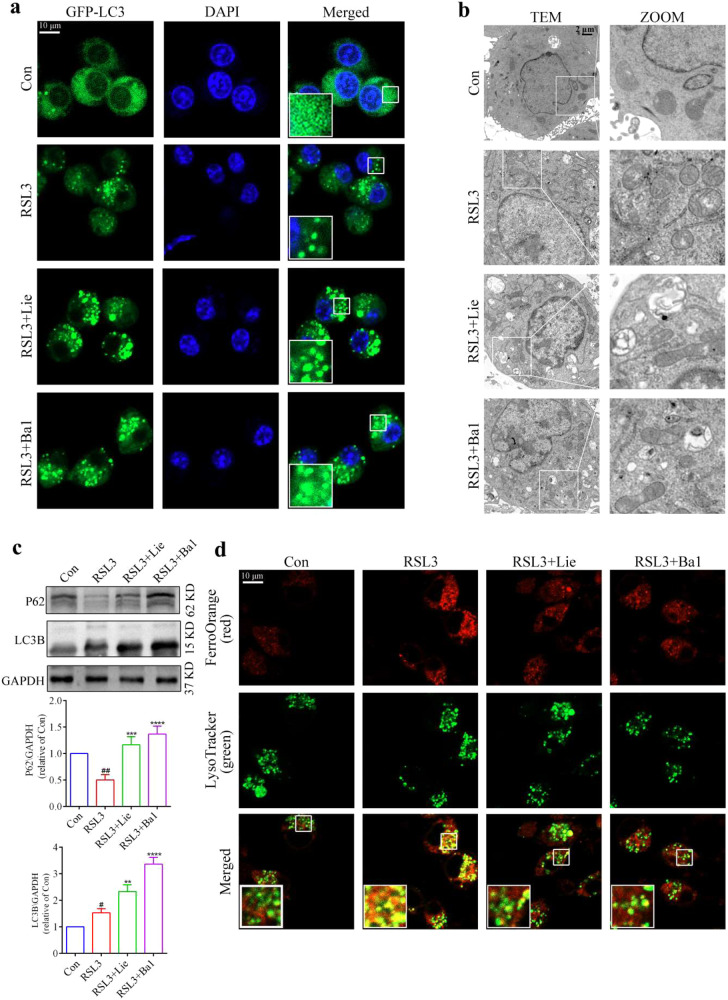
Liensinine inhibits ferritinophagy to inhibit alternatively activated macrophage ferroptosis. **a** EGFP-LC3 spot aggregation was observed under confocal microscopy, Scale bar = 10 µm (*n* = 3). **b** Expression of autophagy-related proteins LC3B-I, LC3B-II, and P62 was detected using western blots. **c** Representative electron micrograph image, Scale bar = 2 µm (*n* = 3). **d** Representative immunofluorescence images of FerroOrange (red) and LysoTracker Green (green) were used to examine the subcellular localization of Fe^2+^ in cells, Scale bar = 10 µm. Results were presented as mean ± SD. (*n* = 3, ^##^*p* < 0.01, ^#^*p* < 0.05, vs. Con. **p < 0.01, ***p < 0.001, ****p < 0.0001, vs. RSL3. Con Control, NS not significant).

We investigated the mechanisms by which Lie inhibits ferroptosis in AAM. Ferroptosis is an iron-dependent mode of cell death [36]. By degrading ferritin, an intracellular iron storage protein, autophagy promotes ferroptosis by releasing stored iron and increasing the quantity of unstable iron in cells [37]. Lie has been found to play a role in autophagy regulation [38]. Owing to the fact that Lie can increase AAM resistance to ferroptosis, we explored whether Lie increases AAM resistance to ferroptosis by modulating ferritinophagy. The laser confocal scanning microscopy analysis revealed increased autophagosome accumulation in the presence of Lie and in the RSL3-induced AAM than in the RSL3 group (Fig. 6a). The ultrastructural alterations in these cells were observed using TEM to further characterize the autophagic properties of AAM following RSL3 and Lie treatment. The autophagic vacuole numbers increased in the Lie + RSL3 group compared to that in the RSL3 group (Fig. 6b). Western blot analysis revealed that Lie significantly enhanced LC3B/GADPH and P62/GADPH expression (Fig. 6c). Owing to the fact that p62 is the link between LC3 and autophagic substrates, autophagy inhibition may correspond with increasing P62 expression levels [39]. Therefore, Lie may be a potent autophagic flux inhibitor, increasing AAM resistance to ferroptosis by suppressing ferritinophagic flux. Using the Fe^2+^-selective fluorescent probe FerroOrange and lysosomal probe LysoTracker Green, we investigated whether Lie could alter the levels of Fe^2+^ in the lysosomes of RSL3-treated AAM. Hence, when AAM was treated with RSL3 combined with Lie, Fe^2+^ levels in the lysosomes (coexistence of FerroOrange and LysoTracker Green) reduced compared to that in the RSL3 group. Bafilomycin A1, an autophagy inhibitor, was used as a positive control, showing almost the same effect as Lie.

### Induction of ferritinophagic flux alterations by liensinine via blocking autophagosome-lysosome fusion in alternatively activated macrophages

**Fig. 7:**
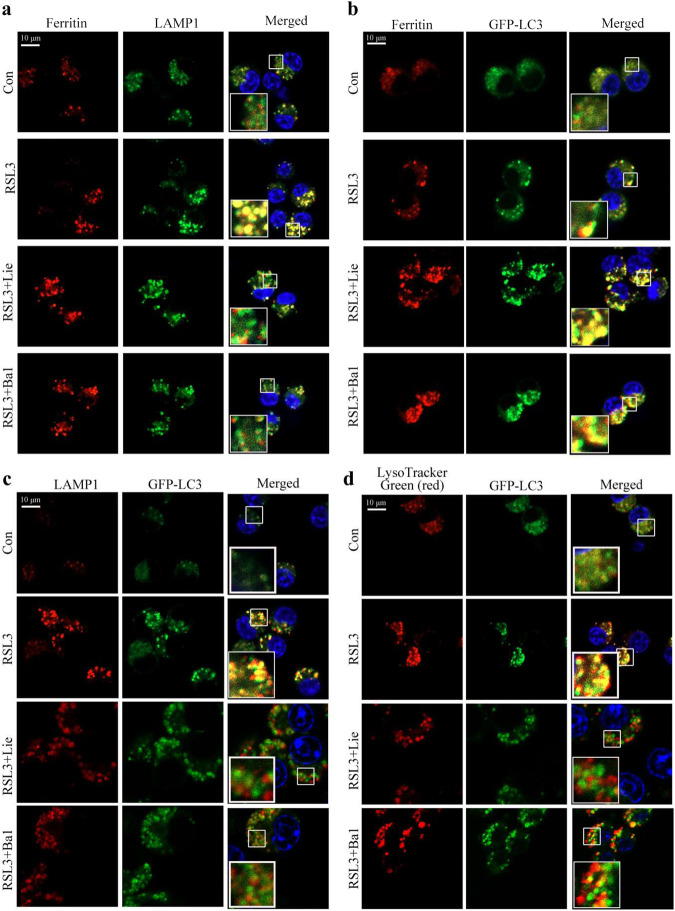
Liensinine induces ferritinophagic flux alterations by blocking autophagosome-lysosome fusion in alternatively activated macrophages. **a** Confocal microscopy images of the co-localization of ferritin (red) with LAMP1 (green), Scale bar = 100 µm (*n* = 3). **b** Confocal microscopy images of the co-localization of ferritin (red) with GFP-LC3 (green), Scale bar = 10 µm (*n* = 3). **c** Confocal microscopy images of immunostained GFP-LC-3 (green) and LAMP1 (red), Scale bar = 10 µm (*n* = 3). **d** Confocal microscopy images of LysoTracker Green (red) and GFP-LC3 (green), Scale bar = 10 µm (*n* = 3).

To determine whether the effects of Lie were due to ferritinophagic flux inhibition, AAM was treated with RSL3 combined with or without Lie, and ferritin punctate co-localization with the lysosomal marker LAMP1 was examined. Ferritin and LAMP1 co-localization was reduced following Lie treatment (Fig. 7a), suggesting that Lie may affect the ferritin autophagosome formation or block autophagosome fusion with lysosomes. We then evaluated LC3 co-localization with ferritin after treating AAM using RSL3 with or without Lie and found that the latter increased LC3-ferritin co-localization (Fig. 7b). These findings imply that Lie had no effect on ferritin autophagosomes formation. Fusing autophagosomes with lysosomes in the late stages of autophagy leads to autophagolysosome formation, and inhibiting this process impairs ferritin degradation. Therefore, we hypothesized that suppressing autophagosome-lysosome fusion could explain the impairment of Lie-induced ferritinophagic flux. To determine whether Lie inhibits the fusion of ferritin autophagosomes with lysosomes, we used immunofluorescence to investigate the co-localization of the autophagosome marker LC3 and the lysosomal membrane marker LAMP1. Lie significantly inhibited LC3-LAMP1 co-localization (Fig. 7c). Similarly, Lie reduced LC3 co-localization and the lysosomal probe LysoTracker was reduced (Fig. 7d), implying that Lie prevented autophagosome-lysosome fusion from inducing impaired ferritinophagic flux; use of bafilomycin A1, an autophagy inhibitor that prevents autophagosome-lysosome fusion, had similar effects (Fig. 7a–d).

## Discussion

ALI is a multi-etiological disease with high morbidity and mortality rates [5]. During ALI progression, Macrophages play critical roles in coordinating tissue destruction and repair following an acute liver injury [12]. However, macrophages are highly malleable and, depending on the stimuli, can develop into phenotypes with diverse properties and effects. Therefore, exerting different regulatory functions on the physiological and pathological activities of the body [13]. Macrophages are mainly polarized into two phenotypes: pro-inflammatory M1 and anti-inflammatory AAM. Many pro-inflammatory cytokines, including IL-1b, iNOS, and TNF-a, are secreted by M1 macrophages, whereas AAM macrophages mainly produce anti-inflammatory factors, such as IL-10, transforming growth factor-b, and arginase 1 [14, 17]. Studies revealed that pro-inflammatory cytokines secreted by M1 macrophages aggravate ALI. In contrast, M2 macrophages were found to enhance tissue damage repair and secrete anti-inflammatory cytokines, facilitating inflammation regression and ALI remission [16–20]. Therefore, increasing AAM numbers may be an effective strategy for ameliorating ALI. Two strategies can be used to increase the number of AAM in ALI: one requires increasing AAM, and the other involves lowering AAM depletion. However, current studies have focused almost exclusively on improving ALI by regulating macrophage reprogramming or increasing the AAM via chemotaxis [11, 14, 17, 20]. Drugs have rarely been reported to ameliorate ALI by lowering AAM depletion and enhancing AAM survival. This could be because drugs that reduce the damage and consumption of AAM also reduce that of M1 macrophages, making it challenging to maintain an equilibrium between the two phenotypes.

M1 macrophages expressing iNOS were recently found to be highly resistant to ferroptosis. However, AAM lacking iNOS expression was highly susceptible to ferroptosis [33]. In il-4-driven macrophage differentiation, loss of Gpx4 activity leads to ferroptotic cell death, but not in M1 macrophages. [40]. Our findings are consistent with those of previous studies. We exposed M1 macrophages and AAM to various cell-death inducers and found that AAM was ferroptosis-sensitive, whereas M1 macrophages were ferroptosis-resistant. Ferroptosis is a new cell death type triggered by excessive Fe^2+^ ion accumulation in the cell [27]. Excessive intracellular Fe^2+^ ion accumulation induces oxidative stress, which promotes lipid peroxidation of cell membranes, protein oxidation, and DNA damage [27]. Clinical studies have revealed that increased hepatic irons reserves and elevated serum ferritin concentrations are typical characteristics of various liver diseases [28]. In vivo studies have shown that iron overload can cause liver damage in mice [41, 42]. In APAP-induced hepatotoxicity, lysosomal iron was also observed to translocate to mitochondria and promote oxidation, and this harmful effect was antagonized by iron chelators [30]. These findings suggest that ferroptosis plays a role in ALI progression. Accordingly, we hypothesized that Fer-1 (a potent ferroptosis inhibitor) could alleviate ALI, possibly rescue ferroptosis, and increase AAM survival in ALI. Our findings confirm that Fer-1 alleviates histopathological changes in the liver and increases the expression of the AAM marker CD206 levels in LPS/D-GalN-treated mice. These findings suggest that ferroptosis is an important factor in ALI pathogenesis, and inhibiting it can partially alleviate LPS/D-GalN-induced ALI while increasing the number of AAM.

Our findings revealed that Fer-1 partially alleviates LPS/D-GalN-induced ALI and increases the number of AAM. However, its limited clinical use is attributed to its in vivo instability. Hence, a more effective drug is needed. The bioactive components of natural products are a significant source of drugs. A practical strategy for developing ALI treatments is the identification of novel medications from herbal monomeric compounds [34]. We identified that Lie effectively inhibits RSL3-induced ferroptosis in AAM after screening the herbal library monomers for small-molecule compounds. Lie was found to reduce lipid peroxides and Fe^2+^ production in RSL3-induced AAM in vitro. These findings suggest that Lie may boost AAM resistance to ferroptosis. In addition, Lie was found to partially protect mice from LPS/D-GalN-induced pathological liver injury and inflammation, attenuate LPS/D-GalN-induced lipid peroxidation, and increase the amount of AAM. Notably, it is hypothesized that the accumulation of iron-dependent lipid peroxidation products is a key factor in the onset of ferroptosis [27]. Further, when the balance between lipid peroxidation product synthesis and clearance are disrupted, such as by inhibiting GPX4, iron-dependent lipid peroxidation product accumulation triggers ferroptosis [29]. 4-HNE is one of the end products of iron-dependent lipid peroxidation and is frequently used as an indicator of ferroptosis [27]. 4-HNE expression was found to be significantly increased in acute liver injury induced by APAP or erastin [30]. In this study, we found that iNOS-positive cells in LPS/D-GalN-treated mice had less 4-HNE than CD206-positive cells and that 4-HNE synthesis in CD206-positive cells was significantly reduced in Lie-treated LPS/D-GalN-induced acute liver injury. These findings suggest M1 macrophages are resistant to ferroptosis in ALI. In contrast, AAM are susceptible to ferroptosis, which may result in an imbalance between M1 macrophages and AAM, and promote the development of intrahepatic inflammation. In addition, the efficacy of Lie to inhibit the hepatic inflammatory response in LPS/D-GalN-treated mice was significantly reduced in macrophage-deficient mice. These findings suggest that Lie inhibits ferroptosis in ALI and is protective in mice against pathological liver injury. Inflammatory responses induced by LPS/D-galactosamine may be partly related to AAM ferroptosis inhibition.

Lie is a bioactive ingredient extracted from lotus seeds that play an important role in preventing and treating various diseases [43]. Moreover, recent studies have revealed that it regulates autophagy. For example, Lie enhances doxorubicin-mediated apoptosis by inhibiting autophagy [44] and suppresses non-small cell lung cancer progression in vitro and in vivo by blocking autophagic flux [45]. Moreover, it preserves beige adipocyte properties by inhibiting mitotic phagocytosis, thereby reducing obesity [46]. To date, the effects of Lie on the relationship between ferroptosis and autophagy have not been well studied. Ferroptosis is an iron-dependent form of oxidative cell death. The Fenton reaction is a chemical reaction that occurs when ferrous iron reacts with hydrogen peroxide to form ferric iron. This results in ROS production, which, if not immediately scavenged, damages lipid membranes and causes cell death [27]. The nuclear receptor coactivator 4, which binds to FTH1 in the autophagosome, transfers the ferritin autophagosome to the lysosome to degrade ferritin and then releases free iron, is an important source of intracellular ferrous ions [36, 47]. Ferroptosis can be prevented by inhibiting ferritinophagy. For example, inhibiting RANKL-induced ferritinophagy can protect osteoclasts from ferroptosis [48], whereas inhibiting ferritinophagy can reduce intracellular iron levels and lipid peroxidation, attenuating ZnONP-induced ferroptosis [49]. Therefore, we hypothesized that Lie might inhibit ferroptosis in AAM by regulating ferritinophagy. Our in vitro experiments revealed that autophagic flux changed following Lie treatment. Further, when AAM was treated with RSL3 and Lie combination, the amount of Fe2+ in the lysosomes was significantly reduced compared to the RSL3 group, suggesting that Lie may inhibit the ferritinophagy flux and reduce Fe2+ levels to increase AAM resistance to ferroptosis. In addition, we found that treating AAM with RSL3 in conjunction with Lie significantly reduced the co-localization of ferritin and the lysosomal marker LAMP1. Nonetheless, the combination did not inhibit the co-localization of LC3 with ferritin. These findings suggest that Lie may attenuate ferroptosis in AAM in ALI by blocking autophagosome-lysosome fusion and inhibiting ferritinophagy.

In conclusion, we revealed that Lie, a novel ferroptosis inhibitor, may inhibit AAM ferroptosis in ALI, maintain the balance between M1 macrophages and AAM, and reduce acute liver damage, potentially by partially inhibiting autophagosome-lysosome fusion and ferritin degradation. Therefore, Lie may be proposed as a novel candidate for treating patients with ALI.

This study had some limitations. We did not identify other potential molecular targets for Lie; hence, whether Lie could bind to multi-target proteins involved in ferroptosis or autophagy remains unknown. In future studies, the following should be considered: (1) the specific target proteins of Lie, (2) mechanisms by which it inhibits ferritinophagy, and (3) whether the findings of this study may be used as a reference point in developing treatments for other types of acute organ injury with comparable pathogenesis to ALI.

## Materials and methods

### Antibodies and reagents

Antibodies against iNOS (ab178945), ferritin (ab75973), LC3B (ab48394), p62 (ab109012), and horseradish peroxidase-conjugated secondary antibodies (ab6721) were purchased from Abcam (Cambridge, MA, USA). Anti-CD206 (24595) antibody was purchased from Cell Signaling Technology (Danvers, MA, USA). An anti-4-hydroxynonenal (4-HNE) (BS-6313R) antibody was purchased from Bioss (Beijing, China); an anti-LAMP-1 (AF4320-SP) antibody was purchased from R&D Systems (Minnesota, USA); and anti-GAPDH (60004-1-1g) antibody was purchased from Proteintech (Wuhan, China). Liensinine (Lie), a TCM library, RSL3, erastin, apoptosis inducers, ferrostatin-1 (Fer-1), and bafilomycin A1 (Ba1) were purchased from Selleck Chemicals (Houston, TX, USA). A necroptosis inducer kit with TSZ was purchased from Beyotime (Shanghai, China). Clodronate liposomes (CLs) were purchased from FormuMax (Shanghai, China).

### RAW264.7 cell culture, polarization, and treatment

RAW264.7 cells were purchased from Procell Life Science&Technology (Wuhan, China) and authenticated by STR profiling. The cells were cultured in high-glucose Dulbecco’s modified Eagle medium (Gibco, USA) with 10% fetal bovine serum (Australian origin; Gibco) and 1% penicillin-streptomycin (Gibco, USA) at 37 °C with 5% CO_2_.

RAW264.7 macrophages were unstimulated to create M0 macrophages. For M1 macrophage creation, RAW264.7 cells were treated with LPS (10 ng/ml; Sigma-Aldrich, St. Louis, MO, USA) and interferon (IFN)-γ (100 ng/ml; PeproTech) for 24 h. For the AAM, RAW264.7 cells were treated with IL-4 (20 ng/ml; PeproTech) for 24 h.

Apoptosis inducer (20 μM, 24 h), pyroptosis inducers (7 μg/ml, 24 h), necrosis inducer (4 x, 24 h), RSL3 inducer (5 μM, 5 h), and erastin inducer (60 μM, 24 h) were used to treat macrophages (M0, M1, and AAM). For 5 h, AAM was treated with RSL3 (5 μM) in the presence or absence of Fer-1 (400 nM). AAM was treated with the 1488 candidates combined with RSL3 (5 μM) for 5 h. AAM was treated with Lie (1, 5, and 10 μM) combined with RSL3 (5 μM) for 5 h. AAM was treated with RSL3 (5 μM) in the presence or absence of Lie (10 μM) for 5 h. AAM was treated with RSL3 (5 μM) in the presence or absence of Lie (10 μM) or bafilomycin A1 (25 nM) for 5 h.

### Cell Counting Kit-8 (CCK-8) and lactate dehydrogenase (LDH) cytotoxicity assays

Cell viability was determined using the CCK-8 kit (Beyotime, Shanghai, China). In addition, LDH levels were measured using LDH Cytotoxicity Assay Kit (Beyotime, Shanghai, China) following the protocol of the manufacturer.

### Propidium iodide (PI) staining

Approximately 1/10th of the medium volume of PI (KGA214-50; Nanjing, China) working solution (20 μM) was added to the culture medium. The cells were incubated at 37 °C for 15 min. DAPI staining was performed for 5 min. The cells were observed under a fluorescence microscope and photographed.

### Transmission electron microscopy (TEM)

Cells or liver tissues were collected and fixed in 2.5% Gluta fixative (Solarbio, Beijing, China) for 30 min in the dark at 25 °C before being stored at 4 °C. Dehydrated samples were embedded in epoxy resin and cut into ultrathin sections. The samples were then examined and photographed using an HT7800 TEM (Hitachi, Japan).

### Glutathione (GSH) and malondialdehyde (MDA) assays

MDA and GSH levels in mouse liver tissues collected were determined using a commercial assay kit (Beyotime, Shanghai, China).

### Divalent iron ion probe detection

FerroOrange working solution (1 mM; Dojindo Laboratories, Kyushu, Japan) was added to the samples and incubated for 30 min before being photographed under a fluorescence microscope.

### Active oxygen determination

Dihydroethidium (Beyotime, Shanghai, China) was used to measure superoxide anion levels in cells and liver tissues. Under different experimental conditions, the cells or tissue sections were placed on coverslips. They were then incubated for 30 min at 37 °C with a 5-µM dihydroethidium working solution before being photographed under a fluorescence microscope.

### Liperfluo assay

Liperfluo (Dojindo Laboratories, Kyushu, Japan) is a Spy-LHP analog that can be used to detect lipid peroxides. Liperfluo working solution (1 mM) was added to the samples and incubated for 30 min at 37 °C before being photographed using a fluorescence microscope.

### Alanine aminotransferase (ALT), aspartate aminotransferase (AST), and inflammatory factor measurements

ALT, AST, and inflammatory factor levels in mice sera were measured with the analytical assay kits. Inflammatory factor assay kits were purchased from Solarbio Science & Technology (Beijing, China). AST and ALT assay kits were purchased from JINGMEI BIOTECHNOLOGY (Suzhou, China).

### Western blotting

RIPA buffer (Beyotime, Shanghai, China) was used to extract proteins from liver tissues. A bicinchoninic acid assay kit was used to determine the protein levels. The proteins were denatured by boiling for 10 min. Protein samples (containing 50 μg per well) were dispensed into the gel and were treated to an initial voltage of 80 V for ~30 min. The voltage was then raised to 120 V for 60 min. After incubation at 26 °C for 2 h, the separated proteins in the gel were transferred onto polyvinylidene difluoride membranes (0.45 μm) and blocked in skimmed milk (5%) at 100 mA/90 min. The corresponding primary antibodies (LC3, 1:1000; P62, 1:1000) were added and incubated overnight at 4 °C. The secondary antibody (1:5000) was then added and incubated for 2 h at 26 °C before being washed four times in TBST for 5 min. The protein bands were then examined.

### Immunohistochemistry and hematoxylin and eosin (H&E) staining

The paraffin-embedded sections were oven dried at 60 °C for 60 min, dewaxed, and then rehydrated. H&E stains were applied for 3 min and 30 s, respectively. Antigen repair was performed under high pressure for 15 min for immunohistochemistry analysis. H_2_O_2_ (3%) in phosphate-buffered saline was applied for 15 min to remove endogenous peroxidase. The tissue was covered with 5% goat serum at 26 °C for 1 h. Dropwise additions of primary antibodies (iNOS, 1:100; CD206, 1:100; 4-HNE, 1:100) were conducted overnight at 4 °C. Fluorescent secondary antibodies (1:200) or horseradish peroxidase-labeled secondary antibodies (1:200) were then added and incubated for 30 min at 26 °C; DAPI staining was used for the fluorescent secondary antibody for 5 min while in the horseradish peroxidase-labeled secondary antibody, staining was continued with diaminobenzidine or tyramide signal amplification for 10 min, followed by hematoxylin re-staining.

### Immunofluorescence

The cells or tissue sections were placed on coverslips. Lysosomes were stained using LysoTracker Red (Beyotime Biotechnology) or LysoTracker Green (Beyotime Biotechnology). The samples were fixed in 4% paraformaldehyde for 30 min, permeabilized for 10 min in 0.1% Triton X-100, then incubated in 10% goat serum for 30 min. LAMP1 (1:100), ferritin (1:100), and 4-HNE (1:100) were added and incubated overnight at 4 °C. The fluorescent secondary antibody (1:200) was then added and incubated for 2 h at 26 °C. The cells were observed using laser scanning confocal or fluorescence microscopy.

### GFP-LC3B transfection

The cells were dispensed into six-well plates at 4 × 10^5^ cells/well at 37 °C with 5% CO_2_ overnight. The old culture medium was aspirated and replaced with a 1.2 ml/well fresh culture medium. Viral solution (20 multiplicity of infection) was added separately. After 24 h, the culture medium was carefully removed and replaced with 2 ml/well fresh complete culture medium before being incubated for 24 h at 37 °C with 5% CO_2_.

### Distribution of ferrous iron in LysoTracker staining

The cells were stained using LysoTracker Red staining working solution (50 nM), then incubated at 37 °C for 20 min. Next, the LysoTracker Red staining working solution was carefully removed, replaced with 1 mol/l of FerroOrange working solution, and incubated for 20 min at 37 °C. After carefully removing the FerroOrange working solution, a fresh cell culture medium was added before examining the samples using a laser confocal microscope.

### Animals and models

Male C57BL/6 mice (6–8 weeks old, weighing 18–23 g) were purchased from Guangdong Experimental Animal Centre (Guangzhou, China). The ALI model was established based on a previous study [50]. Briefly, the mice were intraperitoneally injected with 600 mg/kg of D-GalN (Sigma-Aldrich, Shanghai, China) and 30 μg/kg of LPS (Sigma-Aldrich, Shanghai, China). Mice were treated with different reagents and were randomly assigned to different groups: GalN/LPS + Lie (10, 30, and 60 mg/kg) groups. Mice were administered LPS/D-GalN intraperitoneally to induce acute liver injury before being administered intraperitoneal injections of Lie (10, 30, and 60 mg/kg) 2 h before treatment. Further, survival was monitored over a 24 h period (*n* = 10/group). In the GalN/LPS + Fer1 groups, mice were administered intraperitoneal injections of Fer-1 (10 mg/kg) 2 h before the LPS/D-GalN injection (*n* = 5/group) [51]. In the GalN/LPS + Lie groups, mice were pre-treated with Lie (60 mg/kg) intraperitoneally for 2 h before LPS/D-GalN injections (*n* = 5/group). In the GalN/LPS + Lie + CLs groups, macrophages were depleted by intraperitoneal clodronate liposomes (CLs) injections for 48 h. Mice-depleted macrophages were treated with Lie (60 mg/kg) intraperitoneally for 2 h before being injected with LPS/D-GalN for 6 h (*n* = 5/group). In the control groups, mice were administered saline solution intraperitoneally, whereas Fer-1 was administered intraperitoneally to mice in the Fer1 groups (10 mg/kg). Mice in the Lie groups were administered Lie (60 mg/kg) intraperitoneally. Sera and liver tissues were harvested for analysis 6 h after acute exposure. All animal experiments were approved by the Humane Animal Care Standard and authorized by the Experimental Animal Ethics Committee of Zhongke Industry Holding (Shenzhen) Co., LTD. The individuals conducting the experiments were blinded to the allocation sequence and group allocation.

### Statistical analysis

Each experiment was performed at least three times. GraphPad Prism 7.0 software (GraphPad, San Diego, CA, United States) was used to perform all data analyses. The log-rank test was used to examine survival rates. Unpaired Student’s *t* test was used to compare the two groups. A one-way analysis of variance and Tukey’s multiple comparisons tests were used to compare three or more groups, and statistical significance was set at *p* < 0.05.

## Supplementary information

Supplementary Figure legends

supplemental figure S1

supplemental figure S2

supplemental figure S3

supplemental figure S4

## Data Availability

All data generated or analyzed during this study are included in this published article and its Supplementary Information files. Additional data are available from the corresponding author on reasonable request.
