# Supplementary material for: Role of liensinine in sensitivity of activated macrophages to ferroptosis and in acute liver injury
Source: Cell Death Discov. 2023 Jun 23;9:189. doi: 10.1038/s41420-023-01481-3 (PMC10290152; doi:10.1038/s41420-023-01481-3)
Supplement: Supplementary file 1 — Supplementary Figure legends [file 41420_2023_1481_MOESM1_ESM.docx]

**Supplementary** **Figure legends**

**Fig. S1. Susceptibility of various macrophage types to various forms of cell death.**

(a–d) Resting macrophages (M0): RAW264.7 macrophages were unstimulated; alternatively activated macrophages (AAM): RAW264.7 macrophages were stimulated with IL-4 (20 ng/mL) for 24 h; classical macrophages (M1): RAW264.7 macrophages were stimulated with LPS (20 ng/mL) plus IFN-γ (50 ng/mL) for 24 h. (a) Morphology of RAW264.7 macrophages unstimulated or stimulated with IL-4 or LPS/IFN-γ, respectively, Scale bar = 50 µm, (n = 3). (b–d) IC50 values of the apoptosis inducer, RSL3, erastin, necrosis inducer, and pyroptosis inducer in AAM were determined using CCK8 kit, (n = 3).

**Fig. S2. Fer-1 inhibited the production of the lipid peroxidation marker 4-HNE in LPS/D-GalN-treated mice.**

(a) The treatment group received an intraperitoneal injection of Fer-1 (10 mg/kg) 2 h prior to the LPS/D-GalN injection. Follow-up experiments were performed 6 h after the LPS/D-GalN injections (n = 5/group). The expression of 4-HNE was detected by immunofluorescence in the liver, Scale bar = 100 µm.

**Fig. S3. Liensinine (Lie) against erastin-induced alternatively activated macrophages-ferroptosis *in vitro*.**

(a–g) Resting macrophages (M0): RAW264.7 macrophages were unstimulated; alternatively activated macrophages (AAM): RAW264.7 macrophages were stimulated with IL-4 (20 ng/mL) for 24 h; classical macrophages (M1): RAW264.7 macrophages were stimulated with LPS (20 ng/mL) plus IFN-γ (50 ng/mL) for 24 h.. (a) Morphology of RAW264.7 macrophages (M0, M1, and AAM) unstimulated or stimulated with Lie, Scale bar = 100 µm, (n = 3). (b) After 5 h of stimulation with Lie (10 μM), the expression of iNOS in the M1 macrophages was detected using immunofluorescence, Scale bar = 50 µm, (n = 3). (c–d) After 5 h of stimulation with Lie (10 μM), the expression of CD206 in the M0 macrophages and AAM was detected using immunofluorescence, Scale bar = 50 µm, (n = 3). (e–g) AAM were treated with erastin (60 μM, 24 h) in the presence or absence of Fer-1 (400 nM). (e) PI staining to assess cell death, Scale bar = 200 µm, (n = 3). (f) Live cell fluorescence imaging of AAM to detect lipid peroxide production using Liperfloo, Scale bar = 100 µm, (n = 3). (g) Live cell fluorescence imaging of FerroOrange (red), Scale bar = 100 µm, (n = 3).

**Fig. S4. Liensinine (Lie) inhibited lipid peroxidation and ROS in the livers of mice treated with LPS/D-GalN.**

(a–b) Macrophages were depleted by intraperitoneal injections of clodronate liposomes (CLs) for 48 h in mice. The macrophages-depleted mice were treated with Lie (60 mg/kg) intraperitoneally for 2 h and then injected with LPS/D-GalN for 6 h, after which the liver and blood were removed for subsequent experiments (n = 5/group). (a) Superoxide anion fluorescence detection probe dihydroethidium (DHE) was used to assess the level of ROS in the liver, Scale bar = 100 µm. (b) Expression of 4-HNE was detected by immunofluorescence in liver tissue, Scale bar = 50 µm.
