# Supplementary figures and images for: Role of liensinine in sensitivity of activated macrophages to ferroptosis and in acute liver injury
Source: Cell Death Discov. 2023 Jun 23;9:189. doi: 10.1038/s41420-023-01481-3 (PMC10290152; doi:10.1038/s41420-023-01481-3)

**Figure S1**

**
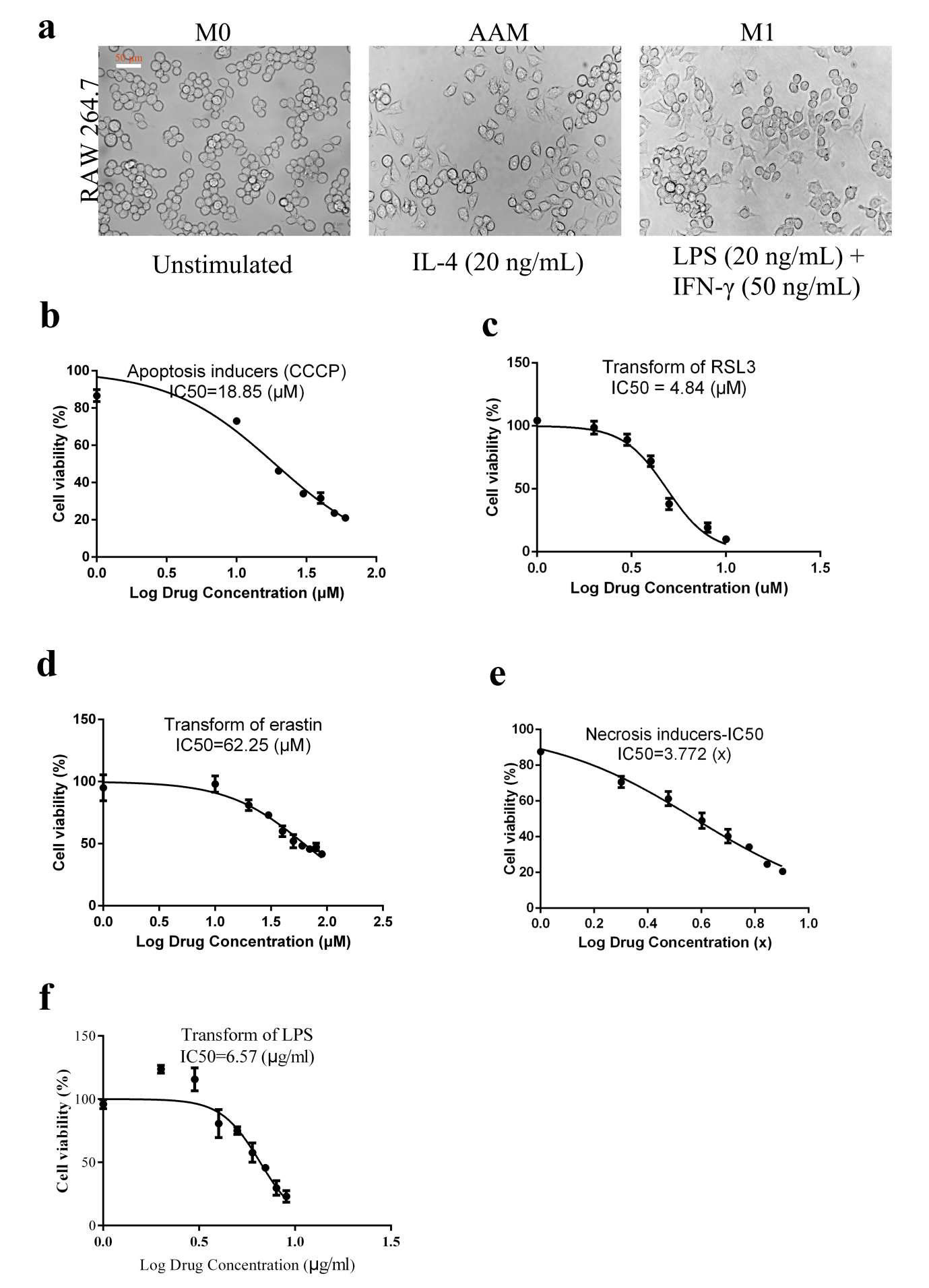
**

Supplement: Supplementary file 2 — supplemental figure S1 [file 41420_2023_1481_MOESM2_ESM.docx]

**Figure S2**

**
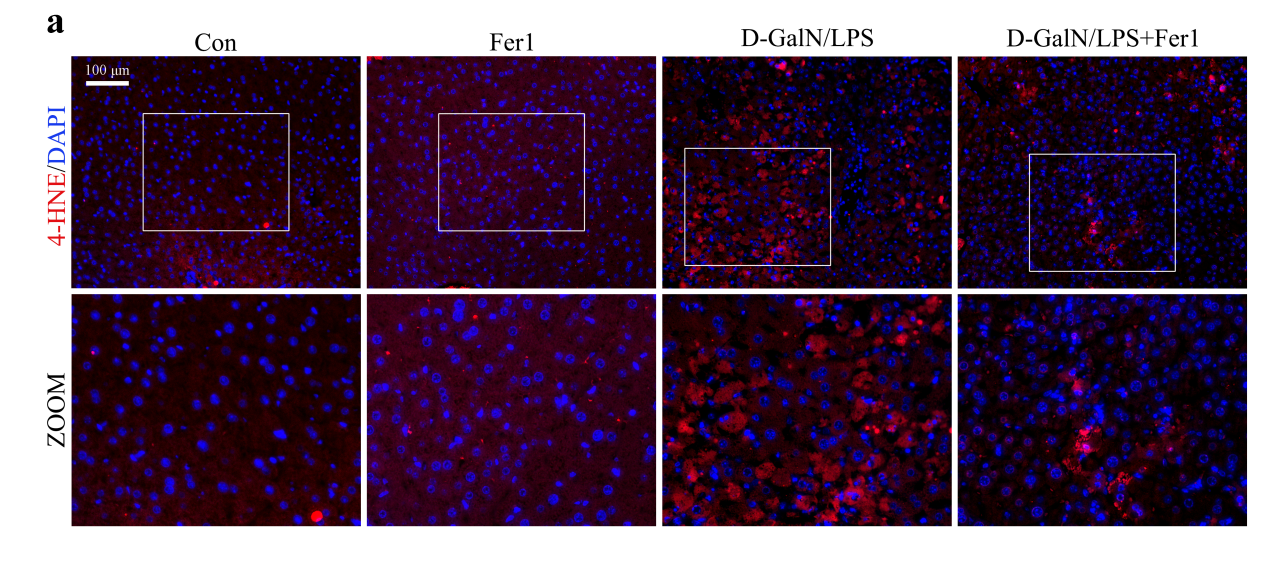
**

Supplement: Supplementary file 3 — supplemental figure S2 [file 41420_2023_1481_MOESM3_ESM.docx]

**Figure S3**

**
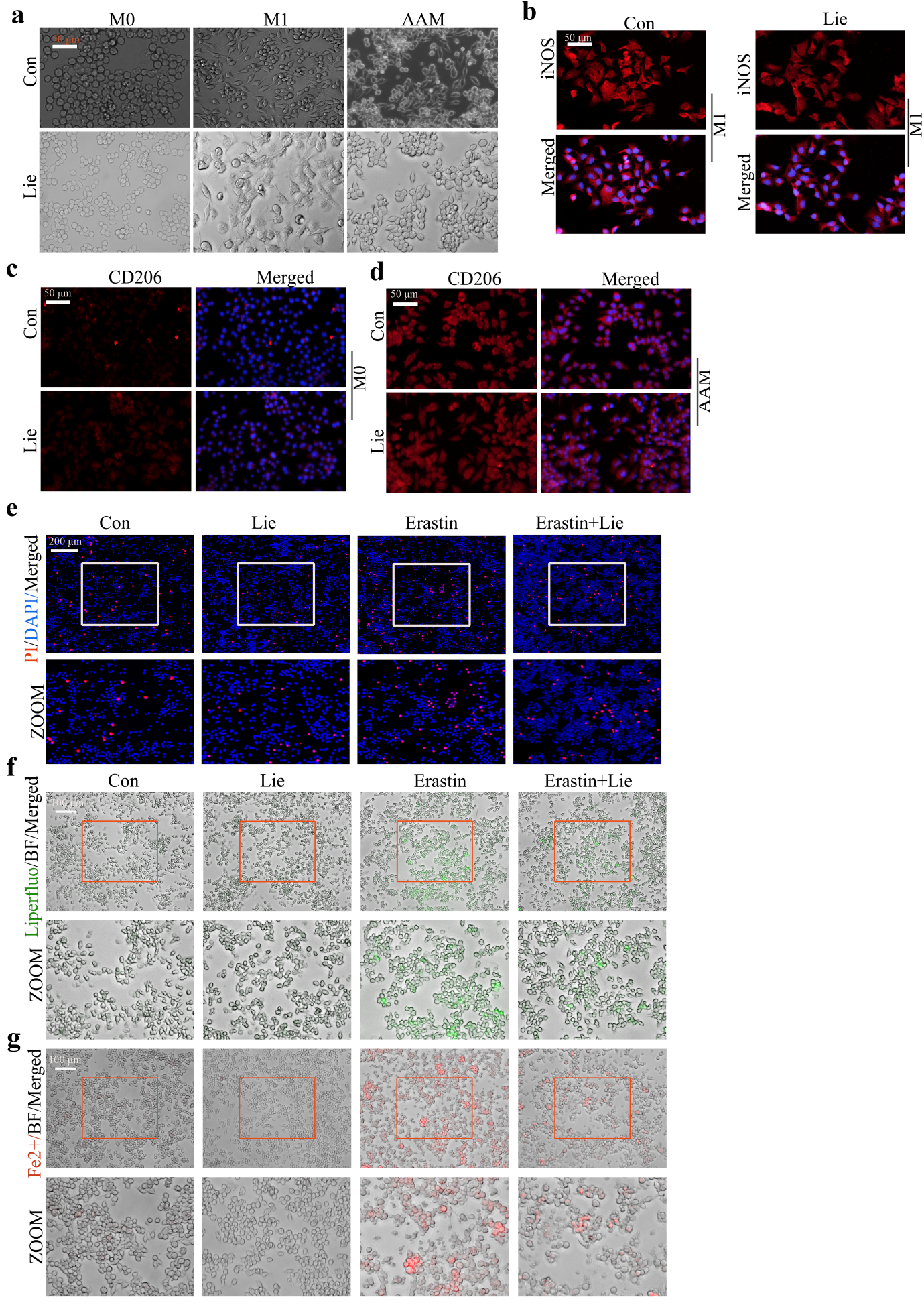
**

Supplement: Supplementary file 4 — supplemental figure S3 [file 41420_2023_1481_MOESM4_ESM.docx]

**Figure S4**

**
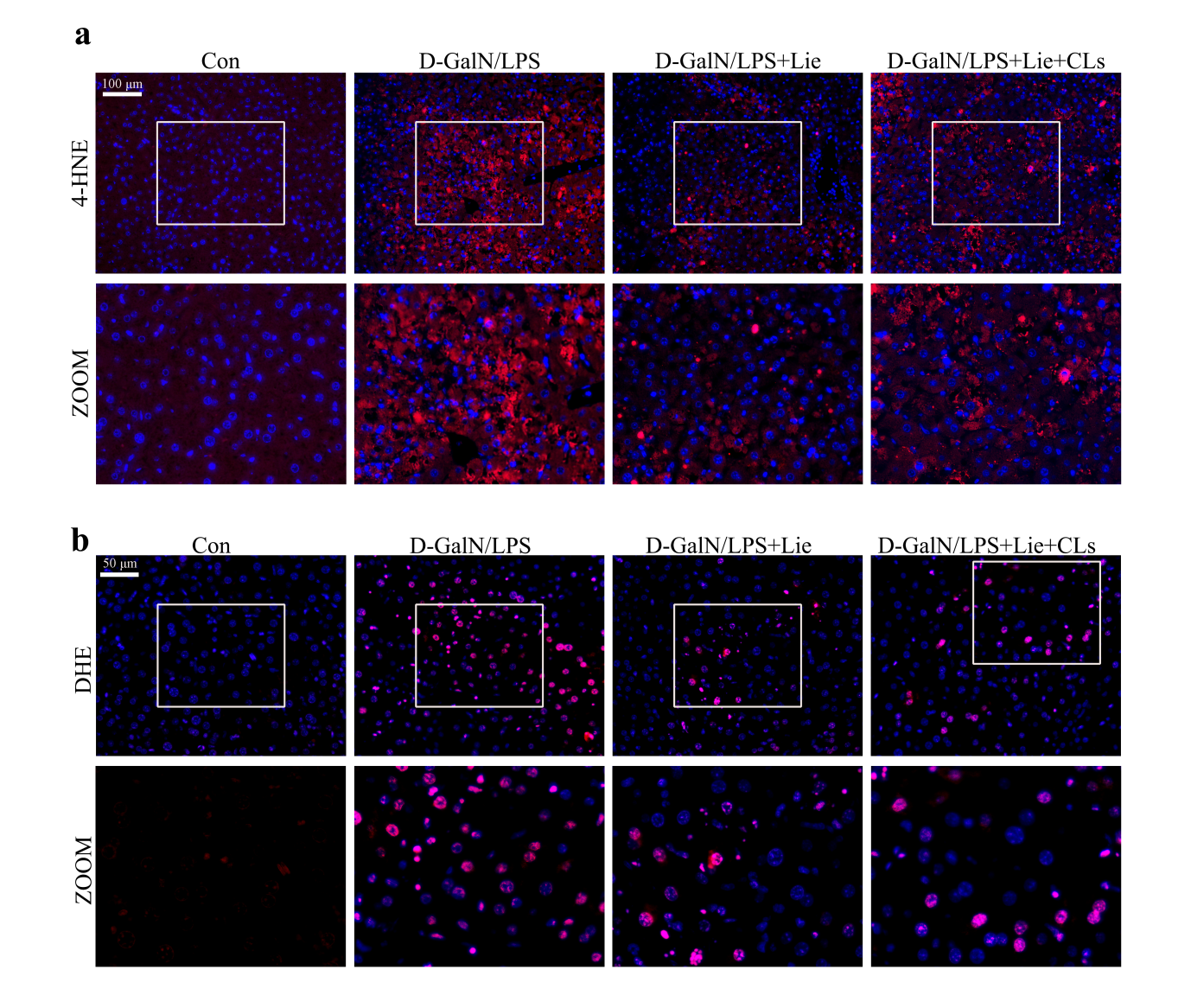
**

Supplement: Supplementary file 5 — supplemental figure S4 [file 41420_2023_1481_MOESM5_ESM.docx]
